# 2D Nanoarchitectures for Sensing/Biosensing Applications

**DOI:** 10.3389/fchem.2022.992793

**Published:** 2022-09-06

**Authors:** Muhammad Asif, Fei Xiao, Mani Govindasamy, Yimin Sun

**Affiliations:** ^1^ Hubei Key Laboratory of Plasma Chemistry and Advanced Materials, School of Materials Science and Engineering, Wuhan Institute of Technology, Wuhan, China; ^2^ Key Laboratory of Material Chemistry for Energy Conversion and Storage, Ministry of Education, School of Chemistry and Chemical Engineering, Huazhong University of Science & Technology, Wuhan, China; ^3^ Department of Materials Engineering, Ming-Chi University of Technology, New Taipei City, Taiwan

**Keywords:** sensors, biosensors, 2D nanomaterials, pollutants, biomolecules, point-of-care

## Abstract

Schematic illustration of 2D materials based sensors for the detection of various analytes.
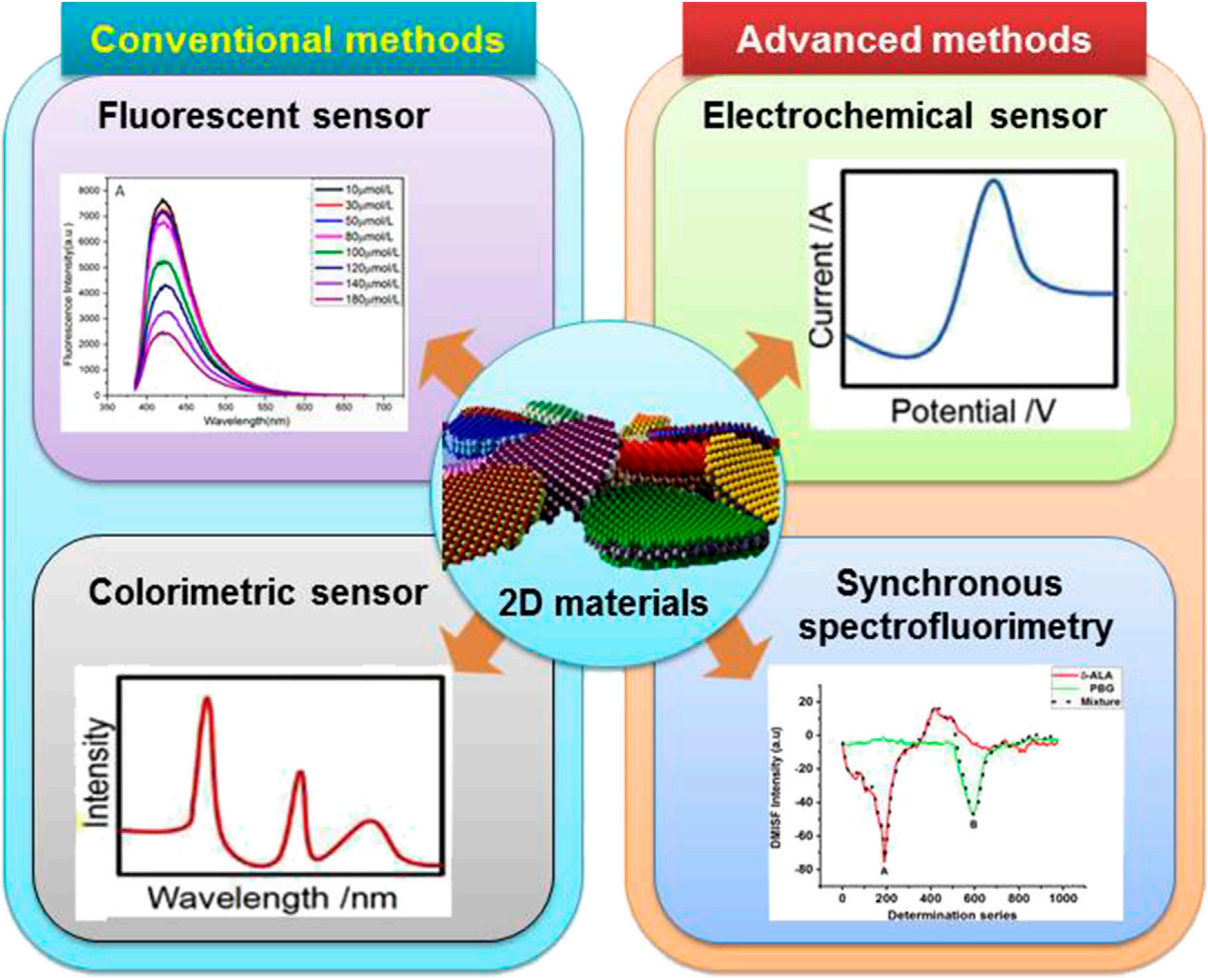

In analytical chemistry, sensing and biosensing are among the most important research areas for carrying out the analysis of a number of target analytes (Hermawan et al., 2021). The growing number of analytes that can be detected using various approaches, along with the large number of sensing platforms that produce analytically beneficial outcomes, highlight the significance of sensing techniques (Asif et al., 2018). Sensing and biosensing techniques are regarded as highly sensitive, specific, and capable of multitasking as shown in Graphical abstract (Zhang et al., 2020).

In comparison to other nanomaterials, 2D nanostructures exhibit encouraging application potential for the *in vitro* detection of small biological molecules, neurotransmitters, and proteins, which in turn are used as biomarkers. These nanostructures are cost-effective and easy to synthesize, and they offer rapid response times in terms of the screening and monitoring of various disorders, including potentially fatal cancers (Iftikhar et al., 2021). They also play a role in improving the sensitivity, selectivity, and stability of the sensing platforms (Aziz et al., 2019). Moreover, 2D nanoarchitectures, when coupled with emerging types of nanostructures and advanced nanotechnology, not only improve sensing capabilities for the real-time detection of gases, pollutants, and small biomolecules but also help these technologies execute *in vitro* and *in vivo* diagnosis (Dral and Johan, 2018). The introduction of point-of-care testing devices, in particular, has revolutionized sensing systems. These devices offer portability, flexibility, non-invasiveness (Asamoah et al., 2021), low cost, time-effectiveness, improved sampling frequency, and implantability (Xu et al., 2019). Meanwhile, trials involving the wireless transmission of analytical data to medical centers for processing have been successful, while the development of ready-to-use and reusable sensors continues to lag behind due to the complexity and high cost of these devices (Asif et al., 2021).

In short, the research field is quite broad, which makes it a bit difficult to represent all the exciting developments in sensing platforms explored in these articles. This Research Topic covers a total of eleven articles, ten of which represent original research and one of which is a review. All articles were contributed by experts in the sensing and biosensing field.

To date, a great deal of effort has been devoted to improving the efficiency and biocompatibility of sensors. In this regard, Wang et al. successfully fabricated a composite material through the polymerization of indole-5-carboxylic acid into poly-5-carboxyindole nanostructures (PI-5-CA), which were further hybridized with carboxylated single-walled carbon nanotubes (C-SWCNTs). The electrode modified with PI-5-CA/C-SWCNTs was used as an electrochemical immunosensor to detect *E. coli* O157:H7, demonstrating a wide linear concentration range of 2.98 × 10^1^ to 2.98 × 10^7^ CFU/ml and a limit of detection (LOD) of 2.5 CFU/ml. Interestingly, the sensor was also used to detect bacteria in water specimens, with decent accuracy.

Colorimetric assays are considered quick and easy to use because they involve a color change phenomenon (Asif et al., 2020). Zhang et al. have developed a rapid and highly sensitive colorimetric method for the detection of L-Histidine (L-His) that uses Cu^2+^ ions, which inhibit the oxidation of the 3,3′,5,5′-tetramethylbenzidine (TMB)-H_2_O_2_ system. The as-constructed sensor showed two linear concentration ranges of 60 nM–1 μM and 1 μM–1 mM, with a LOD of 50 nM, and proved to be efficient for the detection of L-His in urine samples.

Wang et al. fabricated fluorescent copper-based nanoarchitectures of 5–6 nm in size and employed them as sensors for the detection of NO_2_^−^ and temperature. The copper nanomaterials produced fluorescence, which was selectively quenched by nitrite ions. The intensity of the fluorescence also correlated well with temperatures ranging between 20 and 60°C. These nanomaterials are good enough to be used in nanothermometers, devices that, with their high degree of sensitivity and specificity, are considered necessary for, e.g., the detection of dissolved gases in oil, and therefore capable of, e.g., diagnosing faults in power transformers. Keeping this in mind, HYPERLINK Jia et al. constructed a gas sensor based on an Ag_3_-HfSe_2_ monolayer for the detection of C_2_H_4_ and CO molecules and explored their mechanisms and adsorption behaviors. The adsorption effect on C_2_H_4_ was greater than that on CO. The electrical sensitivity of C_2_H_4_ was found to increase up to 55.49% after adsorption, a phenomenon known as chemisorption. This sensor has the potential to efficiently monitor the working status of power transformers.

Tang et al. prepared MnO_2_ nanosheets with glutathione (GSH) to detect Cu^2+^ ions using a simple colorimetric assay. The fabricated sensor exhibited a wide linearity range of 10–300 nM, with an LOD of 6.9 nM, and demonstrated superb anti-interference capability in Cu^2+^ detection from tap water samples. The sensor performed efficiently without any additional H_2_O_2_ or complicated modification processes.

Furthermore, Wu et al. developed a fluorescence sensor for the detection of Bi^3+^ ions. The authors prepared glutathione-protected non-noble transition metal copper nanoparticles (GSH-CuNPs) that carried out their sensing applications via aggregation-induced luminescence. Mechanistically, the fluorescence of the CuNPs was inhibited upon the addition of Bi^3+^ ions. The fabricated sensor was simple, fast, and selective, displaying a linearity range of 0 mmol/L–100 mmol/L, with an LOD of 10 mmol/L. HYPERLINK Ajmal et al.presented a two-step approach, based on derivative matrix-isopotential synchronous fluorescence spectrometry (DMISFS) and the Hantzsch reaction, for the rapid, reliable, and simultaneous detection of δ-aminolevulinic acid (δ-ALA) and porphobilinogen (PBG) in urinary samples. The authors envision their sensor as a novel diagnostic tool for patients with severe abdominal pain and as a potential alternative to the δ-ALA/PBG detection kits currently in use, with both clinical and scientific applications.

Yang et al. discussed the recent progress on electrocatalysts based on metal–organic frameworks (MOFs) in the context of electrochemical sensing applications. In this review, the authors briefly present different types of MOFs-based nanomaterials by classifying them into metal-based, carbon-based, and other MOF-based nanostructures. They also elaborate on the structure–activity–performance relationships between these catalysts and extensively discuss the effects of metal cations and synthetic ligands.

Tong et al. and Huang et al. differentiated between cancerous and normal cells by precisely and accurately detecting H_2_O_2_ via sensors based on an Au nanoparticles-polydopamine-poly acrylic acid-graphene (Au NPs-PDAPAA-graphene) nanohybrid, and on a Cu_2_(OH)_3_NO_3_-wrapped ZnO nanorod assembly [Cu_2_(OH)_3_NO_3_@ZnO], respectively. Owing to the synergistic effects of multicomponent systems, the former electrochemical sensor offered higher sensitivity (LOD of 0.02 μM) than the latter (LOD of 1 μM). Both sensors were successfully employed in real-time tracking of H_2_O_2_ excreted by different types of live cells.

The last contribution to this Research Topic was by Siddique et al. The authors fabricated Cu-ZnO nanorods, which were used in the electrochemical sensing of H_2_O_2_. The constructed sensor exhibited good reproducibility, stability, and selectivity, with a linearity range up to 11 mM and an LOD of 0.16 μM.

In summary, this Research Topic highlights the development of nanomaterials, their structure–activity–performance correlations, their improvements to sensing platforms, their performances, and their various potential applications.

## References

[B1] Asamoah B. O. Uurasjärvi E. Räty J. Koistinen A. Roussey M. Peiponen K. E. (2021). Towards the development of portable and *in situ* optical devices for detection of micro-and nanoplastics in water: A review on the current status. Polymers 13, 730. 10.3390/polym13050730 PubMed Abstract | 10.3390/polym13050730 | Google Scholar 33673495 PMC7956778

[B2] Asif M. Ajmal M. Ashraf G. Muhammad N. Aziz A. Iftikhar T. (2020). The role of Biosensors in coronavirus disease-2019 outbreak. Curr. Opin. Electrochem. 23, 174–184. 10.1016/j.coelec.2020.08.011 PubMed Abstract | 10.1016/j.coelec.2020.08.011 | Google Scholar 32984642 PMC7500281

[B3] Asif M. Aziz A. Azeem M. Wang Z. Ashraf G. Xiao F. (2018). A review on electrochemical biosensing platform based on layered double hydroxides for small molecule biomarkers determination. Adv. Colloid Interface Sci. 262, 21–38. 10.1016/j.cis.2018.11.001 PubMed Abstract | 10.1016/j.cis.2018.11.001 | Google Scholar 30428998

[B4] Asif M. Xu Y. Xiao F. Sun Y. (2021). Diagnosis of COVID-19, vitality of emerging technologies and preventive measures. Chem. Eng. J. 423, 130189. 10.1016/j.cej.2021.130189 PubMed Abstract | 10.1016/j.cej.2021.130189 | Google Scholar 33994842 PMC8103773

[B5] Aziz A. Asif M. Ashraf G. Azeem M. Majeed I. Ajmal M. (2019). Advancements in electrochemical sensing of hydrogen peroxide, glucose and dopamine by using 2D nanoarchitectures of layered double hydroxides or metal dichalcogenides. A review. Microchim. Acta 186, 671. 10.1007/s00604-019-3776-z PubMed Abstract | 10.1007/s00604-019-3776-z | Google Scholar 31489483

[B6] Dral A. P. Johan E. (2018). 2D metal oxide nanoflakes for sensing applications: Review and perspective. Sensors Actuators B Chem. 272, 369–392. 10.1016/j.snb.2018.05.157 10.1016/j.snb.2018.05.157 | Google Scholar

[B7] Hermawan A. Amrillah T. Riapanitra A. Ong W. J. Yin S. (2021). Prospects and challenges of MXenes as emerging sensing materials for flexible and wearable breath-based biomarker diagnosis. Adv. Healthc. Mat. 10, 2100970. 10.1002/adhm.202100970 10.1002/adhm.202100970 | Google Scholar 34318999

[B8] Iftikhar T. Asif M. Aziz A. Ashraf G. Jun S. Li G. (2021). Topical advances in nanomaterials based electrochemical sensors for resorcinol detection. Trends Environ. Anal. Chem. 31, 00138. 10.1016/j.teac.2021.e00138 10.1016/j.teac.2021.e00138 | Google Scholar

[B9] Xu K. Lu Y. Takei K. (2019). Multifunctional skin‐inspired flexible sensor systems for wearable electronics. Adv. Mat. Technol. 4, 1800628. 10.1002/admt.201800628 10.1002/admt.201800628 | Google Scholar

[B10] Zhang R. Belwal T. Li L. Lin X. Xu Y. Luo Z. (2020). Nanomaterial‐based Biosensors for sensing key foodborne pathogens: Advances from recent decades. Compr. Rev. Food Sci. Food Saf. 19, 1465–1487. 10.1111/1541-4337.12576 PubMed Abstract | 10.1111/1541-4337.12576 | Google Scholar 33337098

